# The Relationship Between Knee Osteoarthritis-Related Pain Severity and Daily Activities Involving the Upper and Lower Limbs in Saudi Adults: A Cross-Sectional Study

**DOI:** 10.7759/cureus.48381

**Published:** 2023-11-06

**Authors:** Vishal Vennu, Ali D Al-Otaibi, Saud A Alfadhel, Saad M Bindawas

**Affiliations:** 1 Department of Rehabilitation Sciences, College of Applied Medical Sciences, King Saud University, Riyadh, SAU; 2 Department of Rehabilitation Sciences, College of Applied Medical Sciences, King Saud University, and Physical Therapy Department, Dawadmi General Hospital, Riyadh, SAU; 3 Department of Rehabilitation Sciences, College of Applied Medical Sciences, King Saud University, and Physical Therapy Department, General Directorate of Medical Services, Riyadh, SAU

**Keywords:** climbing, walking, severe pain, daily activity, limb, knee, osteoarthritis

## Abstract

Introduction

Earlier research has shown an association between pain intensity and everyday activities in adults. However, it is vital to examine the relationship within the context of Saudi people who have knee osteoarthritis. Therefore, this study aimed to explore the connection between pain intensity and daily activities involving the lower and upper limbs among patients with knee osteoarthritis in Saudi Arabia.

Methods

This study enrolled 209 individuals aged 55 years and above who were diagnosed with radiographic knee osteoarthritis by physicians from five hospitals in Riyadh, Saudi Arabia, between March 2016 and March 2017. Participants were divided into two groups based on their pain intensity, measured using the visual analog scale. The first group included 141 individuals with mild or moderate pain, while the second group comprised 68 individuals with severe pain. The study assessed the physical functioning of these individuals by evaluating their ability to perform daily activities involving the lower and upper limbs, using the Physical Functioning Subscale of the 36-item Short Form Health Survey, which includes 10 items.

Results

Adjusted logistic regression analysis revealed that individuals experiencing severe pain related to knee osteoarthritis were more likely to encounter difficulties in climbing several flights of stairs (odds ratio [OR] = 1.19, 95% confidence interval [CI] = 1.09-1.29), and one flight of stairs (OR = 1.19, 95% CI = 1.06-1.34), with challenges in bending, kneeling, or stooping (OR = 1.14, 95% CI = 1.05-1.23), walking more than one mile (OR = 1.15, 95% CI = 1.06-1.25), walking several blocks (OR = 1.17, 95% CI = 1.08-1.27), and walking one block (OR = 1.19, 95% CI = 1.06-1.34) than those with mild or moderate pain.

Conclusion

Our study results highlight the significant impact of severe pain on activities like climbing stairs, bending, kneeling, stooping, and walking longer distances among people with knee osteoarthritis in Saudi Arabia.

## Introduction

Knee osteoarthritis (KOA) is a prevalent medical condition characterized by pain and functional limitations [1]. Pain serves as a crucial indicator of KOA and is often associated with the underlying pathology of the disease [2]. The severity of pain in KOA patients can significantly impact their ability to engage in daily activities [3]. Pain-related limitations in daily activities have been identified as a major health concern, contributing to a reduced quality of life, increased disability, and higher healthcare costs among adults with KOA [4,5]. Understanding the relationship between pain levels and daily activities is essential for assessing the impact of KOA on patients' lives.

In Saudi Arabia, daily tasks such as praying, bathing, and eating while seated on the ground require knee flexion, which is deeply ingrained in the local customs and cultural practices [6]. However, KOA pain has emerged as a growing health issue, particularly among individuals aged 50 years and above, leading to significant challenges in knee flexion and limitations in daily activities [4]. Additionally, the influence of environmental and lifestyle factors further contributes to the impact of pain on daily activity limitations [7].

While previous literature has provided evidence of the correlation between pain intensity and daily activities in adults [2,8], it is crucial to examine this relationship in the context of Saudi adults with KOA [9]. Thus, the present study aims to investigate the relationship between pain intensity and daily activities involving the lower and upper limbs among patients with KOA in Saudi Arabia. By comparing the impact of severe pain on lower limb activities (e.g., climbing stairs, bending, kneeling, stooping, walking longer distances) and upper limb activities (e.g., moderate activity, lifting or carrying groceries, bathing or dressing oneself), this study seeks to contribute to the understanding of the functional limitations faced by individuals with KOA. Based on existing literature, it is hypothesized that intense pain will be associated with more significant limitations in both lower and upper limb daily activities among these patients.

## Materials and methods

Study design and setting

A cross-sectional study was carried out between March 2016 and March 2017 in the orthopedic and physical therapy departments of King Saud University Medical City (KSUMC), King Faisal Specialist Hospital & Research Centre (KFSHRC), King Saud Medical City (KSMC), and King Khalid University Hospital (KKUH), Riyadh, Saudi Arabia, as per the Declaration of Helsinki. The ethics committees of the College of Applied Medical Sciences (No: 098-36/37), KSUMC (No: 16/0169/IRB), KFSHRC (No: ORA/1171/37), KSMC (No: H-01-R-053), and KKUH (No: 16/0300/IRB) have approved this study. Written informed consent was obtained from each participant upon enrollment in the study.

Participants

The study involved 209 individuals aged 55 and above, who were diagnosed with KOA through radiography and confirmed by a physician, following the guidelines of the American College of Rheumatology. The study utilized the visual analog scale (VAS) cut-off values [10]; all participants were divided into mild or moderate pain (n = 141) or severe pain (n = 68) groups in approximately a 2:1 ratio, respectively. The ratio of 2:1 was considered more time-efficient and cost-effective based on the primary method for sample size determination in health research [11]. We excluded patients with chronic rheumatoid arthritis, fractures, significant lower limb surgery, or intra-articular injections during the previous six months.

Pain intensity

A single-item continuous VAS of a horizontal or vertical line assessed pain throughout the previous 24 hours during exercise [12]. The pain scale is 10 cm long and ranges from 0 to 10. Zero means no pain, and higher numbers indicate more severe pain. The thresholds for pain classification are 0-4 mm for no pain, 5-44 mm for mild pain, 45-74 mm for moderate pain, and 75-100 mm for severe pain [10]. Participants who reported a VAS score between 5 and 74 mm were classified as experiencing mild or moderate pain, whereas a score of 75 mm or above was indicative of severe pain. It is worth noting that the VAS scale has been deemed a reliable and valid method for measuring pain intensity in adults, as established by previous studies [13].

Daily activities

The 36-item Short-Form Health Survey's 10-item Physical Functioning (10-PF) subscale was used to evaluate the range of daily activities [14]. The 10-PF assesses the degree of health-related limitations in a range of daily activities, such as vigorous activities (item #1) or moderate activities (item #2); lifting or carrying groceries (item #3); climbing multiple flights of stairs (item #4) or just one flight (item #5); bending, kneeling, or stooping (item #6); walking for more than a mile (item #7); walking several blocks (item #8); walking one block (item #9); and bathing or getting dressed (item #10). We categorized all ten items as upper limb movement (items #3 and 10), lower limb movement (items #1, 2, and 6), lower limb walking (items #7, 8, and 9), and lower-limb climbing (items #4 and 5).

Likert's approach for summarizing rating scales was used to score the 10-PF, and the algebraic total of the 10 item scores (1 = very limited; 2 = limited; 3 = not limited at all) was calculated [15]. According to the Likert scale, we further divided it into two levels: not limited and limited, regarding the range of daily activities. The reliability and validity of the 10-PF across patient groups have been well-established elsewhere [16].

Statistical analysis

Data normality was evaluated using the Kolmogorov-Smirnov test [17]. Count and percentage were computed for patients in the two groups for categorical data, while we calculated the mean and standard deviation (SD) for continuous variables. The differences between these groups were demonstrated using the chi-squared test for dichotomized parameters and an independent t-test for continuous variables. We presented the frequency distribution of the range of daily activities based on the pain intensity. A chi-square test or independent Student's t-test was used to determine significant differences between groups.

Unadjusted and adjusted logistic regression models examined the association between radiographic KOA-severe pain and daily activities. The adjusted model included age, sex, education, employment position, body mass index (BMI), and KOA severity. The odds ratios (ORs) and 95% confidence intervals (CIs) were used to present the regression results. The reference used for all analyses was mild or moderate pain.

We analyzed sensitivity to determine the relationship between height and lower-limb climbing. We used a linear regression analysis to examine the association between severe pain and limb walking. Estimates (β) and standard error (SE) were used to present the results from the linear regression. All analyses of the raw data were performed using the Statistical Analysis System (SAS) version 9.4 (SAS Institute, Inc., Cary, North Carolina) for Windows.

## Results

Of the 209 patients enrolled, 141 (67.5%) experienced mild or moderate pain, while 68 (32.5%) had severe pain (Table 1). Most people who reported severe pain (73.5%) were women. The majority of patients with significant pain had only completed elementary school or less (55.9%), had severe OA of the knees (84.6%), and had limited daily activities (32.5 ± 9.1).

**Table 1:** Descriptive characteristics of the total sample stratified by knee pain severity OA, osteoarthritis; BMI, body mass index. *Pearson’s Chi-square test. **Independent Student's t-test.

Characteristics	Mild or moderate pain, N = 141 (67.5%)	Severe pain, N = 68 (32.5%)	χ^2^-value or t-value	p-value^*^
Age (years), mean ± SD	57.6 ± 8.7	59.9 ± 8.1	-1.77	.078^**^
Gender, n (%)				
Male	57 (40.4)	18 (26.5)	3.88	.049
Female	84 (59.6)	50 (73.5)		
Education, n (%)				
Primary school/less	36 (25.5)	38 (55.9)	18.47	.001
High school/more	105 (74.5)	30 (44.1)		
Employment status, n (%)				
Employed	100 (70.9)	54 (79.4)	1.70	.192
Self-employed/retired	41 (29.1)	14 (20.6)		
Affected knee with OA, n (%)				
Right/left	111 (78.7)	58 (85.3)	1.28	.258
Both	30 (21.3)	10 (14.7)		
Knee OA severity, n (%)				
Mild	38 (27.7)	1 (1.5)	77.89	.001
Moderate	72 (52.6)	9 (13.9)		
Severe	27 (19.7)	55 (84.6)		
BMI (kg/m^2^), mean ± SD	32.1 ± 5.5	33.4 ± 5.4	-1.60	.112^**^
Physical function, mean ± SD	46.4 ± 16.6	32.5 ± 9.1	6.44	.001^**^

**Figure 1:**
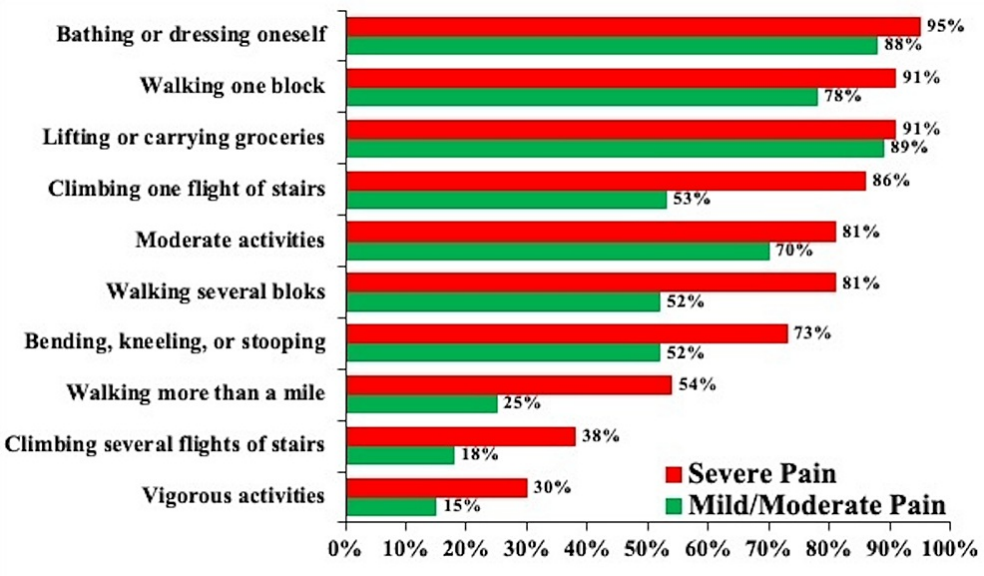
The frequency distribution of a range of daily activities according to pain intensity The significance in frequency distribution between chronic pain (n =68) and mild/moderate pain (n =141) groups was determined using a chi-square test or independent Student's t-test.

Figure 1 presents the frequency distribution of the range of daily activities according to pain intensity. Individuals with KOA-related severe pain had a high-frequency distribution in each daily activity compared to those with mild or moderate pain. Particularly, more than 90% of individuals with KOA-related severe pain had a high-frequency distribution in bathing or dressing themselves (95%), walking one block (91%), and lifting or carrying groceries (91%).

Table 2 presents the association between pain intensity and various daily activities. KOA-severe pain was more likely associated significantly with a range of daily activities by odds of 1.19-fold (95% CI = 1.11-1.27) and 1.18-fold (95% CI = 1.09-1.27) compared to mild or moderate pain after adjusting for age, sex, education, employment status, BMI, affected knee with OA, and KOA severity. Notably, radiographic KOA-severe pain was significantly associated with greater odds of some range of daily activities, such as vigorous activities (aOR = 1.12, 95% CI = 1.02-1.23), climbing several flights of stairs (aOR = 1.01, 95% CI = 1.00-1.20), and one flight of stairs (aOR = 1.19, 95% CI = 1.09-1.29); bending, kneeling, or stooping (aOR = 1.14, 95% CI = 1.05-1.23); walking more than one mile (aOR = 1.15, 95% CI = 1.06-1.25); walking several blocks (aOR = 1.17, 95% CI = 1.08-1.27); and walking one block (aOR = 1.19, 95% CI = 1.06-1.34) after controlling for covariates mentioned above. However, severe knee pain was not likely associated significantly with some range of daily activities, such as moderate activity (aOR = 1.08, 95% CI = 0.99-1.18, p = .069), lifting or carrying groceries (aOR = 0.99, 95% CI = 0.88-1.13, p = .932), and bathing or dressing oneself (aOR = 1.12, 95% CI = 0.98-1.27, p = .101).

**Table 2:** The association between knee osteoarthritis-related severe pain and total and each daily activity involving the upper and lower limbs. OR, odds ratio; aOR, adjusted odds ratio; CI, confidence interval. The participants' total and specific physical function items were evaluated in binary logistic regression (enter method). ^a^Adjusted for age, sex, education, employment status, body mass index, affected knee with knee osteoarthritis, and knee osteoarthritis severity. Reference: Mild or moderate pain.

Dependent variable	Unadjusted OR (95% CI)	p-value	Adjusted^a ^aOR (95% CI)	p-value
Total physical functions	1.19 (1.11-1.27)	.001	1.18 (1.09-1.27)	.001
Upper limb movement				
Lifting or carrying groceries	1.02 (0.91-1.14)	.675	0.99 (0.88-1.13)	.932
Bathing or dressing oneself	1.11 (0.99-1.25)	.083	1.12 (0.98-1.27)	.101
Lower limb movement				
Vigorous activities	1.13 (1.04-1.22)	.004	1.12 (1.02-1.23)	.016
Moderate activities	1.08 (1.00-1.17)	.042	1.08 (0.99-1.18)	.069
Bending, kneeling, or stooping	1.12 (1.04-1.20)	.001	1.14 (1.05-1.23)	.001
Lower limb walking				
Walking more than a mile	1.16 (1.08-1.24)	.001	1.15(1.06-1.25)	.004
Walking several blocks	1.18 (1.09-1.26)	.001	1.17 (1.08-1.27)	.001
Walking one block	1.14 (1.04-1.26)	.005	1.19 (1.06-1.34)	.003
Lower limb climbing				
Climbing several flights of stairs	1.14 (1.06-1.23)	.001	1.01 (1.00-1.20)	.032
Climbing one flight of stairs	1.21 (1.12-1.31)	.001	1.19 (1.09-1.29)	.001
-	R^2^ =0.167; β = -9.27; SE =1.43; p < .001		R^2^ =0.292; β = -7.62; SE =1.43; p < .001	

The impact of height on lower-limb climbing among adults with severe knee pain is shown in Table 3. Height was more likely associated with climbing one flight of stairs by 3.58-fold (95% CI = 1.53-8.39). Even after accounting for all confounders, height still demonstrated a 5.54-fold (95% CI = 1.79-7.89) association with climbing one flight of stairs. Similarly, even after controlling for all covariates, height was 4.58-fold (95% CI = 1.64-8.24) more strongly associated with climbing several flights of stairs.

**Table 3:** The association between height and lower limb climbing among adults with radiographic knee osteoarthritis chronic pain. OR, odds ratio; aOR, adjusted odds ratio; CI, confidence interval. ^a^Adjusted for age, sex, education, employment status, body mass index, affected knee with knee osteoarthritis, and knee osteoarthritis severity.

Variable	Unadjusted OR (95% CI)	p-value	Adjusted^a^ aOR (95% CI)	p-value
Climbing several flights of stairs	3.82 (1.23-11.8)	.020	4.58 (1.64-8.24)	.009
Climbing one flight of stairs	3.58 (1.53-8.39)	.001	5.54 (1.79-7.89)	.001

Table 4 exhibits how severe knee pain affects lower-limb walking. Reduced walking of more than a mile by 0.42 and reduced walking of several blocks by 0.29 were both substantially associated with radiographic KOA-severe pain. Due to radiographic KOA-severe pain, the one-block walking distance was dramatically shortened by 0.20.

**Table 4:** The association between knee pain severity and lower limb walking. β, estimate; SE, standard error. ^a^Adjusted for age, sex, education, employment status, body mass index, affected knee with knee osteoarthritis, and knee osteoarthritis severity.

Variable	Unadjusted β(SE)	p-Value	Adjusted^a ^β(SE)	p-value
Intercept	2.55 (0.15)	.001	13.6 (7.55)	.001
Walking one block	-0.33 (0.09)	.007	-0.20 (0.11)	.087
Walking several blocks	-0.35 (0.10)	.001	-0.29 (0.11)	.008
Walking more than a mile	-0.51 (0.11)	.001	-0.42 (0.14)	.003

## Discussion

This study examined the relationship between pain intensity and daily activities involving the lower and upper limbs among patients with KOA in Saudi Arabia. The results indicated that severe pain related to radiographic KOA was significantly associated with a variety of lower limb activities, even after accounting for age, sex, education, employment status, BMI, affected knee with OA, and KOA severity. For individuals with severe pain related to radiographic KOA, height was a significant factor in lower-limb climbing, particularly when climbing multiple flights of stairs. Walking over a mile, several blocks, and one block were all strongly influenced by radiographic KOA-related severe pain in this group. However, severe pain related to radiographic KOA did not significantly affect certain upper limb activities, such as moderate activity, lifting or carrying groceries, and bathing or dressing oneself, after adjusting for the aforementioned factors.

The present results are consistent with those of earlier studies [15,18]. In particular, the pain associated with stair climbing among community-dwelling older adults with progressive chronic KOA has been reported previously [2]. Clinical and epidemiological research findings have indicated that moderate/severe KOA causes high pain in the leg that affects daily activities [7,19]. Previous comprehensive research found that several physical, demographic (including height), and psychological factors were related to a person's ability to climb stairs while suffering from KOA [20]. However, these studies differed in hypotheses and methodology compared to the current research.

The outcomes of this study align with those of earlier studies [15,21]. In these studies, some range of daily activities, such as moderate activity, lifting or carrying groceries, and bathing or dressing oneself, were not associated significantly with severe pain compared to mild or moderate pain, even after controlling confounders. Moreover, evidence for pain and daily activities in KOA was unclear [9]. A possible explanation for this might be the high heterogeneity across those studies. Another possible reason is that those studies used different populations and methodologies, and the pain was not used as an exposure variable to determine the outcome.

The results from the present study broadly support the work of other studies in which the combination of overweight, arthritis, and other rheumatic conditions showed excellent effects on the objective measure of daily activities [22]. The finding is also in line with our earlier observations, which indicated that severe KOA caused significantly increased pain and reduced health-related quality of life, even after controlling for sociodemographic covariates [4]. This study's results appear to concur with those of a previous study that discovered that the daily activities of older Japanese individuals varied depending on their BMI [23]. That study also demonstrated that men with a high BMI had significantly shorter one-leg standing times with open eyes. Another prospective cohort study revealed that obese adult men were at the highest risk of a decline in daily activities [24]. Our analysis also exhibited gender differences in the risk of lower daily activities. However, this result has not been previously specified.

Strengths and limitations

This study had a strong scientific foundation, with 209 Saudi individuals aged 55 or older who were diagnosed with KOA. They were recruited from multiple hospitals in Riyadh, increasing the sample's diversity. Another strength of this study lies in accurately assessing severe pain and its impact on daily activities. Pain intensity was measured using the VAS, a well-established and validated tool for evaluating pain severity. The study also employed the 10-item Physical Functioning subscale of the 36-item Short-Form Health Survey, which comprehensively evaluates a wide range of lower and upper limb activities. By utilizing these reliable measurement instruments, the study ensured a robust evaluation of the functional limitations associated with severe pain in KOA patients.

The findings of this study shed light on significant associations between severe pain related to radiographic KOA and lower limb activities. The results indicate that individuals experiencing severe pain are more likely to encounter difficulties in climbing stairs, bending, kneeling, stooping, and walking longer distances. These findings provide specific insights into the functional limitations. Saudi adults face KOA and emphasize the need for targeted interventions and effective pain management strategies to enhance their quality of life.

However, it is essential to acknowledge the limitations of this study. First, the cross-sectional design employed in this study restricts the establishment of causal relationships between severe pain and functional limitations. Future longitudinal studies are warranted to understand better the temporal relationship between pain and functional decline in KOA patients. Additionally, the study relied on self-reported measures for pain intensity and daily activities, which may introduce recall bias and might not fully capture the objective functional limitations experienced by the participants.

Study results need to be applicable to a wide range of people. This study only included participants from Riyadh, Saudi Arabia, so results may not apply to other regions or countries. Future studies should aim to include more diverse samples. Also, environmental and lifestyle factors were not considered, which could affect the connection between pain and daily activities. Further exploration is needed.

Implications for research and clinical practice

The findings of this study hold several implications for both research and clinical practice. First, from a research perspective, this study contributes to the existing body of literature by highlighting the specific functional limitations associated with severe pain in individuals with KOA [4,25]. The associations between severe pain and difficulties in activities such as climbing stairs, bending, kneeling, stooping, and walking longer distances provide valuable insights into the impact of pain on daily functioning. These findings emphasize the need for further investigation into the underlying mechanisms linking severe pain and functional limitations in KOA patients.

Moreover, this study underscores the importance of conducting longitudinal studies to elucidate the temporal relationship between severe pain and functional decline in individuals with KOA [26,27]. By following up patients over an extended period, researchers can examine how pain severity and functional limitations evolve and identify potential opportunities for early intervention and preventive strategies. Longitudinal studies can also help establish causality and determine whether effective pain management interventions lead to improved functional outcomes in KOA patients.

From a clinical standpoint, the findings of this study have practical implications for healthcare professionals involved in the management of KOA. The functional limitations of severe pain highlight the need for comprehensive assessment and tailored treatment plans [28]. Healthcare providers should prioritize the evaluation of pain intensity and its impact on specific daily activities to gain a holistic understanding of patients' functional impairments. This information can guide the development of personalized interventions that target pain management and aim to improve patients' functional abilities and overall quality of life.

The study's findings also emphasize the importance of implementing multidisciplinary approaches in clinical practice [29]. Given the complex nature of severe pain and its impact on functional limitations, a collaborative effort involving healthcare professionals from various disciplines, such as rheumatology, physical therapy, and occupational therapy, can provide comprehensive care for individuals with KOA. Integrated treatment plans incorporating pharmacological interventions, physical therapy exercises, assistive devices, and patient education on self-management strategies may yield better outcomes in pain reduction and functional improvement.

When designing interventions, healthcare providers must take into account the sociocultural context and individual needs of their patients. To ensure effectiveness and relevance for their unique patient population, clinicians should customize interventions accordingly. It is important to note that a study conducted in Riyadh, Saudi Arabia, may not accurately represent the diverse demographics and cultural factors in other regions or countries.

## Conclusions

This cross-sectional study provides valuable insights into the relationship between severe pain and daily activities involving the upper and lower limbs among patients with KOA in Saudi Arabia. The findings reveal that radiographic KOA-related severe pain significantly impacts lower limb activities, such as climbing and walking. In contrast, no significant association was observed with upper limb activities. This emphasizes the need for tailored exercise regimens targeting lower limb functions to improve functional outcomes and quality of life for patients experiencing KOA-related severe pain. The study highlights the importance of developing rehabilitative approaches that address the unique challenges faced by these patients. Research should focus on finding effective exercise protocols and assessing their long-term impact on reducing pain and improving functional abilities in patients with KOA. This information can help healthcare professionals create interventions that combat functional limitations caused by severe pain.
